# Essential Annotation Schema for Ecology (EASE)—A framework supporting the efficient data annotation and faceted navigation in ecology

**DOI:** 10.1371/journal.pone.0186170

**Published:** 2017-10-12

**Authors:** Claas-Thido Pfaff, David Eichenberg, Mario Liebergesell, Birgitta König-Ries, Christian Wirth

**Affiliations:** 1 Department of Special Botany and Functional Biodiversity, University of Leipzig, Germany; 2 German Centre for Integrative Biodiversity Research (iDiv) Halle-Jena-Leipzig, Germany; 3 Department of Mathematics and Computer Science, Friedrich Schiller University of Jena, Germany; Mayo Clinic Arizona, UNITED STATES

## Abstract

Ecology has become a data intensive science over the last decades which often relies on the reuse of data in cross-experimental analyses. However, finding data which qualifies for the reuse in a specific context can be challenging. It requires good quality metadata and annotations as well as efficient search strategies. To date, full text search (often on the metadata only) is the most widely used search strategy although it is known to be inaccurate. Faceted navigation is providing a filter mechanism which is based on fine granular metadata, categorizing search objects along numeric and categorical parameters relevant for their discovery. Selecting from these parameters during a full text search creates a system of filters which allows to refine and improve the results towards more relevance. We developed a framework for the efficient annotation and faceted navigation in ecology. It consists of an XML schema for storing the annotation of search objects and is accompanied by a vocabulary focused on ecology to support the annotation process. The framework consolidates ideas which originate from widely accepted metadata standards, textbooks, scientific literature, and vocabularies as well as from expert knowledge contributed by researchers from ecology and adjacent disciplines.

## Introduction

Technological progress is driving the efficient acquisition, the dissemination and the reuse of data in ecology. Today data is created at an increasing pace and large research networks are used to provide access to ecological data for a broad audience [1,2]. With an improved access to a wide range of ecological data many potential benefits arise. It can help to reduce the amount of redundant data acquisition efforts or facilitate the formation of new collaborations. The reuse of data in fact has become one of the most important strategies in contemporary ecological synthesis projects (e.g. NCEAS: [3,4]). It is not only a basis for reproducible science but also a precondition for synthesizing knowledge. Data reuse allows to extend the scope of studies in order to cover wider temporal and spatial scales which are relevant to human societies and which help to generalize theory across environmental contexts. For example, meta-analyses reusing data from scattered experiments have allowed to develop the theory of multifunctionality in biodiversity/ecosystem functioning research [5], and extended functional biodiversity research from plots to continents [6] and the parameterization of global climate models [7]. Although the reuse of data is important for research in ecology it can be challenging to find suitable data which qualifies for the reuse in a specific context and which comes with context data necessary for an integration into meta-analyses.

The essential prerequisites for an optimal reuse of ecological data are detailed metadata, annotations and efficient search strategies [8,9]. Metadata standards which are used in the context of ecology cover a broad range of information (e.g. [8]). They deal with topics like the temporal and spatial extent of data, or the organisms and methods covered by a study [8,10]. This information is typically provided in large detail using full text descriptions which then can serve as a basis for a full text search. A full text search basically matches strings which are given in a search box with strings that are contained in data and metadata (e.g. abstracts, method descriptions, variable names). A full text search, however, comes with several idiosyncrasies which are reducing its effectiveness. For example, it is typically not aware of synonyms or homonyms nor does it account for broader, narrower or closely related terms relevant for a specific search term. On top of that a full text search lacks the understanding of the semantic meaning of a search query and thus often fails to provide satisfactory results [11,12]. As an example: Searching for the keyword “Carbon” using a full text search across an ecological database will potentially yield a host of results. This might include results from global change studies using elevated *carbon* dioxide concentrations as experimental treatment, soil survey reporting *carbon* concentrations in the subsoil, paleoclimate studies employing *carbon* isotope discrimination in tree rings, or field observations near *Carbon Village* in Alberta, Canada.

Faceted navigation is a mechanism which is frequently used in combination with full text search as it allows a refinement of the search to improve the results. As prerequisite a faceted navigation requires the search objects (e.g. datasets, pictures or products) to be classified in categories. This classification can be done along an arbitrary amount of categories. However, the categories are often reflecting the inherent characteristics of the search objects. In e-commerce that means for example the price, the type or the brand of a product whereas in ecology, for example, the name of the study regions, authors or information related to time and date are potential categories. The categorization is typically stored as an annotation which in turn is stored as sidecar file with the search object [13]. A selection from the categories during a search can then incrementally build up a filter which is restricting the results to match the selected criteria. With respect to the example mentioned above: If the full text search on “Carbon” is complemented by a faceted navigation based on a classification which is using “experimental treatment” and “variable name” as categories then the information can be used to separate the results selecting one or the other explicitly depending on the requirements (e.g. picking “experimental treatment = elevated *carbon* dioxide concentration”).

While the general mechanism of a faceted navigation is simple, the main challenge remains in defining useful parameters and vocabulary which are relevant for the classification of the search object to allow for an efficient discovery [14]. In the context of the German Federation for Biological Data (GFBio, http://www.gfbio.org) and in close collaboration with the German Center for Integrative Biodiversity Research (iDiv) Halle-Jena-Leipzig we set out to define requirements for an efficient annotation of ecological data optimized for a faceted navigation discovery. We screened various sources of information like metadata standards, textbooks, scientific literature and vocabularies to search for useful patterns and concepts suitable for an annotation of ecological data.

Here we present a framework that we call the Essential Annotation Schema for Ecology (EASE) consisting of two parts. The first part is an annotation schema which is based on XML Schema Definition (XSD). It allows to store the information about the classification of search objects along several categories serving as a basis for a faceted annotation and navigation application. The XML schema is accompanied by a vocabulary with a focus on ecology which provides support for the annotation through the provision of ecologically relevant conceptual keywords. The framework is a synthesis which consolidates ideas that originate from expert knowledge, widely accepted metadata standards, and ecological theories and concepts (e.g. used to structure content in textbooks), scientific literature and standardized vocabularies. In the following we present the framework and the underlying design principles and provide an outlook towards a tool based on the framework supporting time efficient annotations and the faceted navigation for an improved discovery and reuse of ecological data.

## Project context

GFBio has the goal to bundle available cyber infrastructure in Germany in order to support researchers in biology and ecology along the whole life cycle of data. GFBio thus aims at supporting the planning of new projects, the acquisition and analysis of data, the publication process, the curation of data and metadata as well as the long term storage of data. Finally, the GFBio web portal will serve as a central point of reference in Germany for the access to biological data including advanced search and features to foster the reuse of biological data and the collaboration between researchers. In order to support the development of the EASE framework several (10 in total) workshops have been set up in close collaboration with the German Centre for Integrative Biodiversity Research (iDiv) Halle-Jena-Leipzig. Domain experts from ecology and adjacent disciplines have been invited to contribute their ideas formulating general design principles for the framework and to discuss and drive the development of the vocabulary.

## Design principles

As a first step, design principles have been defined to set up the general guidelines for the development of the EASE framework.

### Parsimony

In order to support a time efficient annotation, the framework should be kept as simple as possible in regards of structure and the content. This optimization, however, should be done carefully by still maintaining a differentiated and consistent description of ecological data. An example: Time represents an important aspect in ecology which is typically covered by calendar dates and times. Larger time frames are covered by numerical references (e.g. 18 Mio years ago) or by named geological time periods. The International Chronostratigraphic Chart (ICC) is an effort which aims to define the geological time frames of earth history. It defines eons (5 in total: Phanerozoic, Precambrian, Proterozoic, Archean, and Hadean), eras (10 in total: e.g. Cenozoic, Mesozoic, Paleozoic), periods (22 in total: e.g. Quaternary, Neogene, Paleogene), epochs (34 in total: e.g. Holocene, Pleistocene, Pliocene) and ages (98 in total: e.g. Calabrian, Gelasian, Piacenzian). The time frames are getting more granular from eons to ages and the fine granular time frames are nested in the larger ones. For simplicity of the framework and the annotation process it could be argued to ignore e.g. “ages” or at least make them optional. While this would sacrifice some granularity, it would simplify the annotation and still provide a consistent classification depicting the larger temporal context.

### Comprehensiveness

Despite the fact that the framework is striving for parsimony it also has the goal to achieve comprehensiveness. EASE aims at defining essential orthogonal dimensions according to which ecological content can be precisely described. Comprehensiveness is not accomplished by using many different, but rather a few and strictly complementary dimensions. This is reflected by using broad domain relevant topics which are covered in the annotation schema (e.g. time: start time and end time, space: name of locations, method: general approach of the study) but also by the quality how the topics are covered in detail. As an example: Understanding processes and mechanisms is an important aspect to many ecological studies. Thus, the annotation schema contains a part dealing with ecological processes. The processes are covered in a certain breadth asking not only for the name of the process itself but also for related aspects like the objects which are involved (e.g. Organisms, Chemical, Matter, and Energy) and for a generic characterization of the process (e.g. Uptake, Release, and Exchange). The vocabulary is providing a list of widely used and well defined ecological processes which supports the annotation process providing suggested content for the process name field in the schema. As the number of processes used in ecology is potentially endless a list has been designed covering widely used and well defined generic processes e.g. demography (i.e. death, birth, growth), disturbances (e.g. windstorm, fire) or interactions (e.g. parasitism, mutualism).

## The framework

### Vocabulary

Several workshops were carried out comprising in total 35 researchers from ecology and adjacent disciplines. Top level categories for the framework have been collected and eight categories were finally selected. These top level categories represent orthogonal dimensions of information in the search space relevant in ecology (e.g. time, space, methods). In the workshops the selected top level categories have been substantiated in a top-down approach defining a vocabulary with increasing detail. Additional material such as textbooks [15–17] and standardized vocabularies (e.g. World Reference Base for Soil Resources: http://www.fao.org/soils-portal/soil-survey/soil-classification/world-reference-base/en/, International Chronostratigraphic Chart: http://www.stratigraphy.org/index.php/ics-chart-timescale) have been reviewed in order to find useful conceptual keywords and patterns for the annotation framework. The vocabulary of the framework is detailed below along the selected top level categories. The complete framework is available on GitHub (https://git.io/v1Vty) and the sections below are containing references to the according parts of the vocabulary hosted online.

- Time

This is the facet of EASE which captures temporal aspects relevant for ecology. It includes the start and the end of a data acquisition, geological time frames as well as the temporal resolution and extent of the study. The dates and times in EASE are conform to ISO8601 and names of time zones follow the IANA time zone database (http://www.iana.org/time-zones). The geological time frames refer to those given in the International Chronostratigraphic Chart (ICC) which defines and names time ranges in order to express the time scale of earth history (http://www.stratigraphy.org/index.php/ics-chart-timescale). For the temporal extent and the temporal granularity, the vocabulary contains categories along common units of time e.g. “Second”, “Minute”, “Hour”, and “Day” (c.f. vocabulary https://git.io/v1Vtd). In a faceted discovery that ultimately allows to select for data which is matching a desired temporal resolution. For example, studies interested in a fine seasonal resolution typically search for data carried out over at least a whole year with measurements taken on a daily or hourly basis (e.g. atmospheric temperature measurements).

- Space

The space facet of the EASE framework deals with information related to localities and regions. It captures the names of locations, the location type as well as the hierarchical relation of a location to countries and continents. For the location type as well as for the countries and the continents the EASE vocabulary provides predefined lists. They are containing e.g. “City”, “Stream”, and “Lake” (c.f. vocabulary https://git.io/v1sA1) for location types or names of countries and continents like “Andorra”, “Afghanistan”, “Africa”, “Asia” and “Europe” (c.f. vocabulary https://git.io/v1sAS) which has been incorporated from the GeoNames ontology (http://www.geonames.org/). In addition to such explicit definitions of locations, the EASE framework allows to specify a bounding box as well as the exact study site coordinates. The bounding box provides a coarse localization using decimal degree values. The coordinates are captured using the Universal Transverse Mercator (UTM) and the World Geodetic System 1984 (WGS84) datum. Similar as in the time facet the space facet provides a resolution and an extent. To this end the vocabulary provides predefined categorical values being “Point” (<1 m^2^), “Plot” (1 m^2^–0.01 km^2^), “Region” (0.01 km^2^–10000 km^2^), “Continent” (10000 km^2^–100000000 km^2^) and “Global” (larger) (c.f. vocabulary https://git.io/v1Vtj). This allows to filter for data which comes with the desired spatial resolution and extent. For example, data that has been gathered at the landscape scale (exceeding 10 km^2^) but within which several localized study plots were established where measurements have been taken.

- Sphere

**Fig 1:**
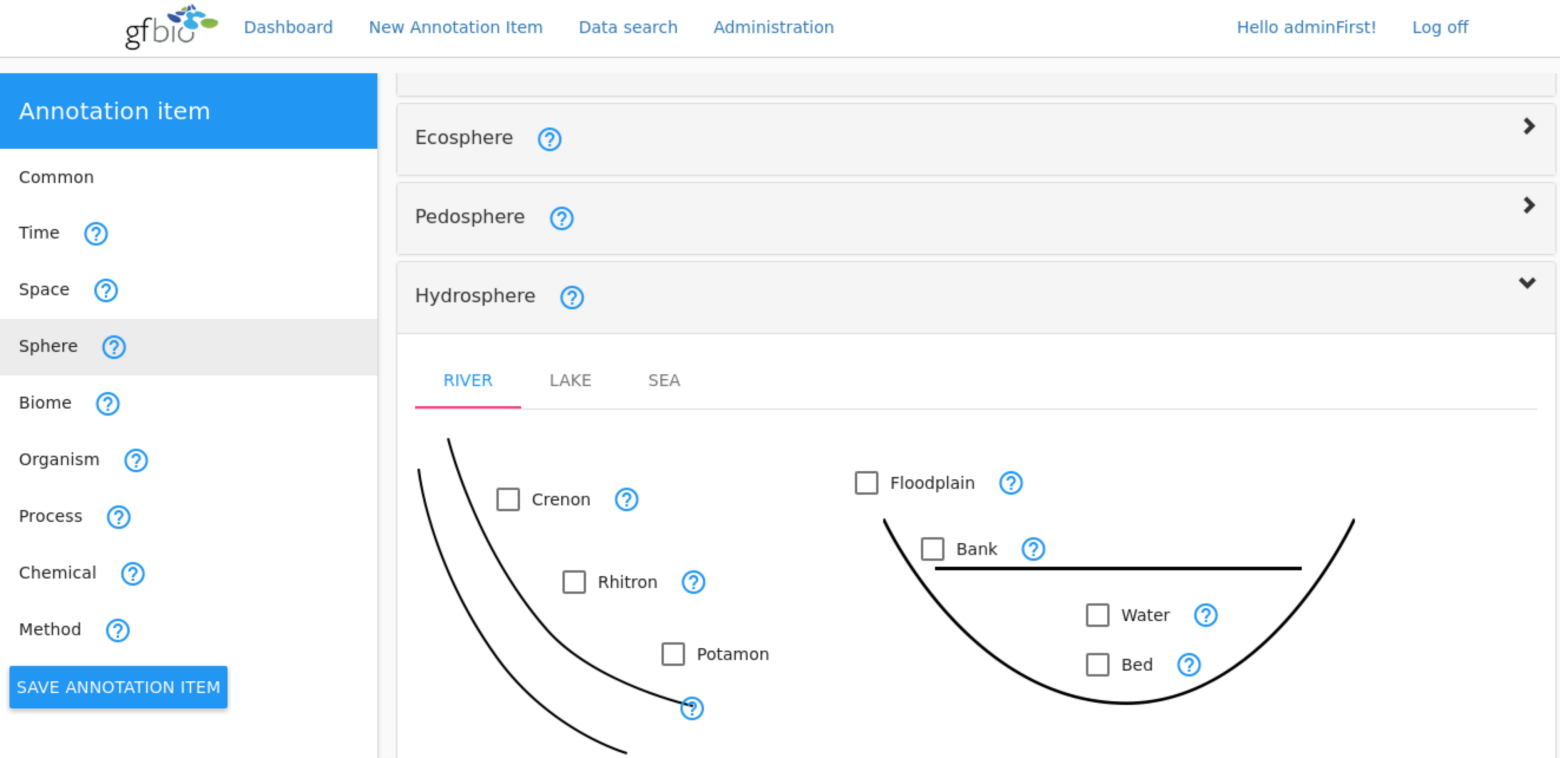
A mockup of a potential annotation tool which will be developed in the future based on the principles of the EASE framework. The figure here specifically depicts the sphere part, detailing the sub-facet hydrosphere. To allow for a finer granularity within the hydrosphere, the tool will allow to specify specific zones in and related to rivers, lakes or the sea. Within these sub-facets, one can easily state where measurements or samples have been taken. To guide the user and allow for a qualitative annotation, definitions of the respective concepts should be displayed e.g. by resting with the cursor over the question marks. In case the user does not find suitable concepts in a specific category he will be provided with an option to extend the annotation vocabulary on the fly (i.e. not shown here).

The sphere part comprises aspects of the pedosphere, the hydrosphere, the atmosphere and the lithosphere. It complements the spatial information of the EASE framework covered in the location facet by identifying compartments and vertical layers within ecosystems or larger spatial reference units. For example, it allows to specify a distinct layer within the atmosphere (e.g. Troposphere, c.f. vocabulary https://git.io/v1OUU) or a layer within a body of water (e.g. Abyssopelagic, c.f. vocabulary https://git.io/v1OUI) to state where the data has been gathered. Apart from this, the sphere facet also captures the levels of biological organization. For that purpose the vocabulary provides predefined categories ranging from the “Atom” over “Cell” and “Organ” up to the “Biosphere” (c.f. vocabulary https://git.io/v1Of7). This finer level of granularity in faceting allows in the end for the selection of data which focuses on a specific organizational level or which comes from a specific compartment in the biosphere like a certain layer in the atmosphere or the soil. Fig 1 shows an example how the annotation could look like with a potential user interface. Based on the definitions given in the vocabulary, annotation (and search) can be achieved by ticking the matching category provided by the tool.

- Biome

The biome facet of EASE captures relevant aspects which describe biomes. This comprises the latitudinal (e.g. Boreal, Temperate, Tropic, c.f. vocabulary: https://git.io/v1OU4) and altitudinal zonation (e.g. Nivale, Montane, c.f. vocabulary: https://git.io/v1OUE), the moisture regime (e.g. Humid, Arid, c.f. vocabulary: https://git.io/v1OUi), the continentality (e.g. Continental, Maritime, c.f. vocabulary: https://git.io/v1OUX) as well as the physiognomy of the biome (e.g. Savannah, Shrubland, c.f. vocabulary: https://git.io/v1OUD) [18]. Below these higher levels of information, the EASE framework also extends into more specifics which are dealing with oro- and pedobiomes, as well as elevation and edaphic features. The vocabulary provides conceptual keywords for selection which are containing e.g. “Amphibiome”, “Halobiome” or “Helobiome”(c.f. vocabulary: https://git.io/v1OfJ). The biome part also deals with the classification of biomes comprising their general condition with “Natural” or “Urban” and their dominant form of usage with e.g. “Agriculture”, “Forestry” or “Fishery” (c.f. vocabulary: https://git.io/v1OvN). It is important to note that many of these features are difficult to infer from the location alone because the fine-scale heterogeneity of hydrography, soil types, physiognomy and land-use is not appropriately resolved in digital maps.

- Organism

The organism facet of EASE deals with the scientific names and taxonomy of organisms. The schema captures scientific names separately for botanical, zoological, fungal organisms and for viruses). For the taxonomy of organisms, the schema of EASE is containing elements named along the main ranks of the Linnean topology which are “Domain”, “Kingdom” (e.g. Plantae, Animalia), “Division” (botany) or “Phylum” (zoology), “Class”, “Order”, “Family” and “Genus”.

- Process

**Fig 2:**
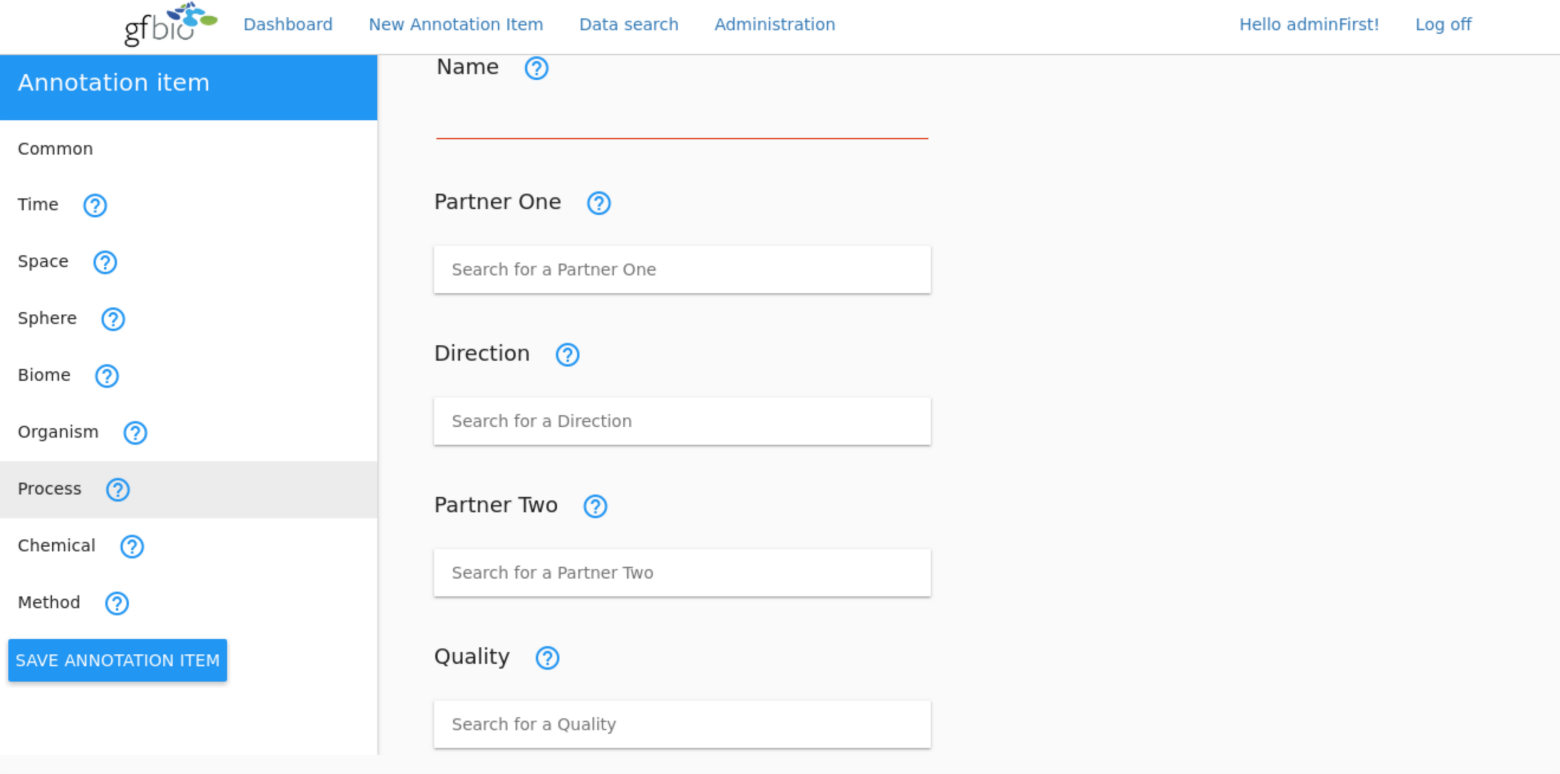
A mockup of a potential annotation tool which will be developed in the future based on the principles of the EASE framework. The figure here specifically depicts the interaction part of processes. It allows to specify the interaction name, the partners, the direction and the quality of the interaction. For the free input fields like the name of the interaction here in this part of the annotation tool auto completion functionality will be provided. This allows to pick from suggestions during the annotation which come from the EASE vocabulary. If a user however is not able to find the right conceptual keyword the vocabulary could be extended creating a new term as required and adding it to the list of annotation terms to be reused by others.

The process facet deals with relevant aspects of ecological processes. To this end the vocabulary supports the annotation by providing a generic list of ecological processes which comprises e.g. the “Adaption”, “Speciation” and “Migration” (c.f. vocabulary: https://git.io/v1OfZ). Additionally the process part deals with interactions, where the user is presented with the option to specify the interacting partners based on kingdoms (e.g. “Plantae”, “Animalia”), the direction of the interaction (“Mutual”, “Affects”, “Is Affected By”) and the quality of the interaction (e.g. “Amensalism”, “Antagonism” c.f. vocabulary: https://git.io/v1OfE). Not only does this allow to select a particular process in the end but also to carry out a search for interaction process related datasets in a very generic way. For example, one can select all data that deals with the interaction between fungi and plants where the direction from the first to the second interaction partner is specified as “Affects” with the quality being “Antagonistic”. That in the end would select data dealing with fungi as plant parasites but not as symbionts (see Fig 2).

- Chemical

The chemical facet deals with all aspects of chemistry being part of ecological data. This comprises chemical elements and compounds which have been measured as well their function in the biological context. The vocabulary here supports the annotation by providing a list of elements based on the periodic table as well as a list of chemical compounds and classes of compounds e.g. “Lipids”, “Carbohydrates”, “Amino Acids” (c.f. vocabulary: https://git.io/v1OfT) which has been compiled from various sources [16,17,19]. Moreover, the biological functions of chemicals which are relevant in ecological studies are covered by conceptual keywords like e.g. “Antibody”, “Attractant” or “Repellent” (c.f. vocabulary: https://git.io/v1OfY) which has been inspired by parts from the Chemical Entities of Biological Interest ontology (CHEBI) (http://www.ebi.ac.uk/ols/ontologies/chebi).

- Method

The methodological facet of the EASE framework captures the general approach and the context of the study. The vocabulary provides a list of generic approach types being either “Virtual” (e.g. simulation), “Manipulative” (i.e. with experimental factors mostly controlled) or “Observational” (i.e. where plot selection creates factor gradients) (https://git.io/v1OfK). The context of the study approach is captured by categories like “Microcosm” (e.g. lab experiment), “Mesocosm” (e.g. ecotron, greenhouse experiment) to “Macrocosm” (e.g. field studies) (https://git.io/v1Ofi). On top of that the method part of EASE captures the variables that either have been manipulated in a study. The vocabulary provides a list of aspects which are manipulated frequently to form gradients containing conceptual keywords like e.g. the “Producer diversity”, the “Consumer density” or the “Nutrient availability” (https://git.io/v1OfD).

### Schema

In parallel to the development of the vocabulary detailed above the EASE XML Schema has been created to serve as foundation for an annotation and faceted navigation application. It is built using the XML Schema Definition (XSD) standard. In order to discover structures suitable for reuse in the annotation schema we screened three XML based metadata standards which are frequently used in the context of ecology (see also S1–S5 Tables). These were:

1. Darwin Core (version: 2015-06-05) which is a standardized metadata schema maintained by the members of the Biodiversity Information Standards (TDWG). It started as a loose collection of terms with a clear semantic meaning. The focus of DwC is to capture and to exchange detailed information about organisms. Darwin Core is separated into nine main topics, six of which are dealing with information like the acquisition event of data, locations, and the geological context, the occurrence of organisms and their taxonomy and the authority of identification. The other parts of the schema deal with general context information which comprises the names and addresses of institutions as well as the nature of the data record [10].
2. The Ecological Metadata Language standard (EML, version 2.0.0) is developed and maintained by the Knowledge Network for Biocomplexity (KNB). It is an initiative with the goal to provide a sophisticated metadata standard for ecology. It has a modular and flexible design which allows using specific parts while neglecting others depending on the use case. It has four top level modules which represent resources that can be described. This comprises dataset, literature, software and protocol. The schema defines a host of modules which allow to capture detailed information about the resources (e.g. Access Rights, Physical Aspects: e.g. File format; Related Parties: e.g. associated people and organizations; Time and Organism related aspects: e.g. Time frame, Taxonomy) [8].
3. The Access to Biological Collection Data (version 2.06) is a metadata standard for the access and the exchange of data about specimens in collections and observations. It is used by the Global Biodiversity Information Facility (GBIF) and the Biological Collection Access Service for Europe network (BioCASe: [20]). The schema is strongly hierarchically organized capturing e.g. aspects about biotopes, specimen, data acquisition events and contacts (e.g. authors, institutes) as well as a detailed history about the location of physical collection objects (https://github.com/tdwg/abcd).

All of the schemas equally well cover aspects of time and space as well as methods and organisms which are essential for a description of data in ecology (see also S1–S5 Tables). The EASE schema provides a well-organized structure for an efficient annotation in ecology which is revolving around the eight facets of the vocabulary detailed above. Apart from that the schema it also defines elements which store general information like responsible parties (e.g. contact and author names and addresses), a reference to the hosting data center, the title and the abstract of the search object and information about how to access the data (e.g. URL, file path, database id). The schema has been designed with an application in mind which is supporting the future maintenance and growth of the vocabulary. Thus the schema allows to store new conceptual keywords not only including their scientific definition but with their associated Unique Resource Identifier (URI) which also provides a link to external vocabularies like ontologies or thesauri [9].

## Discussion

Metadata which is associated with ecological data today is often utilized to support full text search [9]. Although full text search has seen some improvements over time it comes with several immanent issues which often lead to unsatisfactory search results [11]. Faceted navigation is a strategy which gained much popularity over the last decade and by today is successfully applied in a multitude of applications ranging from e-commerce to science [13]. While the basic principle of facets is simple the main challenge remains in the design of the classification attributes [13]. They require a careful design adapted to the specific use case and in order to reflect not only the bare characteristics of a resource but also the requirements of the searching user. The existing metadata schemata that we reviewed for the design of the schema were already covering many aspects we needed in fine detail which have been reused in the structure of the EASE schema (e.g. time and date from EML [8] and organism related aspects of ABCD) but many other detailed aspects have been developed during the workshops based on discussions revolving around particular user needs (e.g. simple temporal and spatial extent and resolution of data or detailed interactions). Next to appropriate attributes which capture information about the search object a vocabulary which is supporting the annotation is equally important.

There are basically two opposing strategies for the provision of a vocabulary. The first follows a top-down approach, where the developer of the annotation schema creates a fixed hierarchy and finite list of terms. The advantage of this approach is that the resulting vocabulary does clearly focus on the essential dimensions and terms. However, top-down designed vocabulary is likely to be incomplete compared to real user requirements. The second strategy is a bottom-up approach like it is known from social tagging [21]. There users are allowed to freely tag their resources (e.g. pictures, datasets). The resulting pool of keywords forms an unstructured vocabulary which is called a folksonomy [22]. This strategy can be very powerful. It is easy to use even without any prior knowledge about a specific vocabulary or annotations and the vocabulary can flexibly grow to reflect the interests and the needs of a user community. However, maturing folksonomy are likely to inflate quickly accumulating redundancy e.g. in form of synonyms, spelling mistakes and different language terms referring to the same semantic concept and they are also likely to contain highly personalized tags which are hard to understand and reuse for others [23].

With the EASE framework we set out to strike a balance between the methods mentioned above. In the creation of the ecological annotation vocabulary we started with a top down approach which is based on a multitude of standards, textbooks and expert knowledge. In the schema we do stick to the top down approach forcing the user to pick from a limited set of vocabulary options for many of the annotation attributes (content restricted attributes). This is especially true where frequent changes of vocabulary are unlikely (e.g. time zones, countries, continents) or where the vocabulary reflects a finite and use case specific gradient (e.g. temporal and spatial resolution). However, there are other parts in the schema which are more open and basically follow a combined approach. There, some vocabulary is provided as an option to pick from but they are not exclusively restricted to these terms which allows the vocabulary to grow (e.g. names of processes, the chemical compounds and the names of variables used as gradients in a study). However, the growth of the vocabulary in these elements should not be uncontrolled. An application on top of the schema should subject new vocabulary to a curation process which (i) ‘harvests’ the emerging new concepts and (ii) and allows a curator to incorporate them in their original or a modified form into the backbone of the EASE vocabulary in order to prevent the problems we see arise with folksonomies.

In the near future we aim to develop an application based on the EASE framework and the mockups we have shown (GitHub: https://git.io/v5wWe). It will provide features which allow for the efficient and fast annotation of data in ecology. It will come with an auto completion so it is possible to pick from meaningful suggestions during the annotation. If a user should not be able to find an appropriate term for the annotation, the tool will help to create vocabulary on the fly and then subject the new created concepts to a curation process. The application will provide support for the annotation of data in a single and batch mode and allow to create annotation templates which then can be applied to any amount of data to speed up the annotation process which is e.g. useful with data coming from the same project (some aspects are not changing). The tool will also integrate with a set of carefully selected external services to provide further vocabulary resources e.g. to fuel the suggestion mechanism beyond the EASE basic vocabulary (e.g. the GFBio terminology service https://www.gfbio.org/data/annotateandconnect). Here it is important to note again that the EASE annotation schema allows storing the URIs of terms used in an annotation. This enables a path to all the content and the knowledge which is modelled in external vocabularies and it allows to link resources described via EASE with many other resource even if they have not been described with EASE. For example, when we pick an environment from the ENVO ontology (e.g. soil) during the annotation in EASE and store the URI this allows us to query and compare all resources which use terms from ENVO for the annotation no matter of the annotation format (e.g. search for datasets which contain soil related parameters).

With the EASE framework we provide a basis for a detailed and highly organized annotation of ecological data which allows to situate data in the ecological search space. The framework can serve as a starting point for new projects and can help them to maintain a harmonized vocabulary facilitating data discovery with a faceted navigation. At the moment the EASE vocabulary is a simple controlled vocabulary. However, the combination of the schema, the vocabulary and the future application together provide a potential platform which allows communities of ecologists to produce and agree on a useful folksonomy which later on can be harvested as raw material for the creation of more elaborate ontologies [24]. Our framework is highly compliant with the topics that are covered by widely used metadata standards in ecology. Thus it is straight forward and easy to ingest information about resources already described via metadata in form of EML, ABCD, or DwC (S1–S5 Tables). The extendibility of the framework can potentially provide new insights increasing the knowledge in metadata sciences and allow a fine granular control over the yield of results combined with a full text search for a better discovery of data in ecological databases.

## Supporting information

S1 Table It shows the conceptual topics of time in EASE in relation to how the topics are covered in EML, ABCD and DwC metadata standards (X = not explicitly available as element in the schema). This mapping also provides an idea on how future ingestion of information from the schemata to EASE can be implemented e.g. using XSLT transformations.
(DOCX)

S2 Table It shows the conceptual topics for space in EASE in relation to how the topics are covered in the EML, ABCD and DwC metadata standards (X = not explicitly available as element in the schema). This mapping also provides an idea on how future ingestion of information from the schemata to EASE can be implemented e.g. using XSLT transformations.
(DOCX)

S3 Table It shows the conceptual topics for biomes in EASE in relation to how the topics are covered in the EML, ABCD and DwC metadata standards (X = not explicitly available as element in the schema). This mapping also provides an idea on how future ingestion of information from the schemata to EASE can be implemented e.g. using XSLT transformations.
(DOCX)

S4 Table It shows the conceptual topics for organisms in EASE in relation to how the topics are covered in the EML, ABCD and DwC metadata standards (X = not explicitly available as element in the schema). This mapping also provides an idea on how future ingestion of information from the schemata to EASE can be implemented e.g. using XSLT transformations.
(DOCX)

S5 Table It shows the conceptual topics for methods in EASE in relation to how the topics are covered in the EML, ABCD and DwC metadata standards (X *=* not explicitly available as element in the schema). This mapping also provides an idea on how future ingestion of information from the schemata to EASE can be implemented e.g. using XSLT transformations.
(DOCX)
