# Supplementary material for: Essential Annotation Schema for Ecology (EASE)—A framework supporting the efficient data annotation and faceted navigation in ecology
Source: PLoS One. 2017 Oct 12;12(10):e0186170. doi: 10.1371/journal.pone.0186170 (PMC5638456; doi:10.1371/journal.pone.0186170)
Supplement: S1 Table — This mapping also provides an idea on how future ingestion of information from the schemata to EASE can be implemented e.g. using XSLT transformations. (DOCX) [file pone.0186170.s001.docx]

| EASE | EML | ABCD | DwC |
| --- | --- | --- | --- |
| Time range for a data acquisition with ISO conform start and end date and the time zone (Olson time zone names) | Time range for a data acquisition with ISO conform start and end date (coverage module) | **X** (But a time frame capturing a collection unit identification event) | Time range of a data acquisition event |
| Geological time frames (International Chronostratigraphic Chart) | Time ranges with an alternative time scale in the coverage module | Geological time frames along bio-, chrono- and litho- stratigraphy | Geological context with upper and lower boundaries specifying a geological time frame |
| Temporal extent (second, minute, …) | **X** | **X** | **X** |
| Temporal resolution (second, minute, …) | **X** | **X** | **X** |
