# Supplementary material for: Essential Annotation Schema for Ecology (EASE)—A framework supporting the efficient data annotation and faceted navigation in ecology
Source: PLoS One. 2017 Oct 12;12(10):e0186170. doi: 10.1371/journal.pone.0186170 (PMC5638456; doi:10.1371/journal.pone.0186170)
Supplement: S2 Table — This mapping also provides an idea on how future ingestion of information from the schemata to EASE can be implemented e.g. using XSLT transformations. (DOCX) [file pone.0186170.s002.docx]

| EASE | EML | ABCD | DwC |
| --- | --- | --- | --- |
| Location name and type (e.g. River, Ocean) as well as the hierarchical relation to a country and continent (GeoNames) | **X** (But potentially the location names and the relation information can be provided as full text geographic description in the coverage module) | Location name and hierarchical relation as well as a way to specify close by locations | Location name and type in form of specific elements (e.g. island = xxx, country = xxx) , Hierarchical relation of the location |
| Bounding box and elevation as well as coordinates | Bounding box in decimal degrees, elevation and complex polygons | **X** (But has a field which allows to specify a download URL for polygon information) | Arbitrary complex polygons in “Well-known Language” markup format |
| Spatial extent (point, plot, …) | **X** | **X** | **X** |
| Spatial resolution (point, plot, …) | **X** | **X** | **X** |
