# Supplementary material for: Essential Annotation Schema for Ecology (EASE)—A framework supporting the efficient data annotation and faceted navigation in ecology
Source: PLoS One. 2017 Oct 12;12(10):e0186170. doi: 10.1371/journal.pone.0186170 (PMC5638456; doi:10.1371/journal.pone.0186170)
Supplement: S3 Table — This mapping also provides an idea on how future ingestion of information from the schemata to EASE can be implemented e.g. using XSLT transformations. (DOCX) [file pone.0186170.s003.docx]

| EASE | EML | ABCD | DwC |
| --- | --- | --- | --- |
| Parameterized biome information e.g. latitudinal and longitudinal zonation, water availability and physiognomy. | **X** (But potentially can be provided as full text description in the geographic coverage) | **X** (But captures the Biotope in the context of gathering a collection unit) | **X** (But captures the Habitat in the context of a data acquisition event) |
| Condition of biome and land use type | **X** | **X** | **X** |
