# Supplementary material for: Essential Annotation Schema for Ecology (EASE)—A framework supporting the efficient data annotation and faceted navigation in ecology
Source: PLoS One. 2017 Oct 12;12(10):e0186170. doi: 10.1371/journal.pone.0186170 (PMC5638456; doi:10.1371/journal.pone.0186170)
Supplement: S4 Table — This mapping also provides an idea on how future ingestion of information from the schemata to EASE can be implemented e.g. using XSLT transformations. (DOCX) [file pone.0186170.s004.docx]

| EASE | EML | ABCD | DwC |
| --- | --- | --- | --- |
| Taxonomy restricted to elements along the main ranks of the Linnean topology. Scientific species names are captured separately for fungi, viruses, plants, and animals | Taxonomy with a free to specify rank and value for the taxon | Taxonomy with free to specify higher taxon name of the organism. Scientific species names are captured separate for fungi, viruses, plants, animals | Taxonomy along the elements of the main ranks of the Linnean topology and free to define taxonomic classification (e.g. Animalia, Chordata) |
