# Supplementary material for: Essential Annotation Schema for Ecology (EASE)—A framework supporting the efficient data annotation and faceted navigation in ecology
Source: PLoS One. 2017 Oct 12;12(10):e0186170. doi: 10.1371/journal.pone.0186170 (PMC5638456; doi:10.1371/journal.pone.0186170)
Supplement: S5 Table — This mapping also provides an idea on how future ingestion of information from the schemata to EASE can be implemented e.g. using XSLT transformations. (DOCX) [file pone.0186170.s005.docx]

| EASE | EML | ABCD | DwC |
| --- | --- | --- | --- |
| General study approach by type and localization | **X** (But allows to specify detailed step by step method protocols in the methods module) | **X** (But a way to describe a method used to make a collection or observation) | **X** (But a description about the measurement methods e.g. a reference to a protocol) |
| Variables by name and, a modifier that designates if they have been measured or modified | Variables and units and a direct link to tabular data allowing also the detailed description of categories in data | A generic way to specify a measurement or fact including information like e.g. a date and time and a unit of the measurement | The name of a variable, the accuracy and the unit of a measurement |
